# The NMT Scalp EEG Dataset: An Open-Source Annotated Dataset of Healthy and Pathological EEG Recordings for Predictive Modeling

**DOI:** 10.3389/fnins.2021.755817

**Published:** 2022-01-05

**Authors:** Hassan Aqeel Khan, Rahat Ul Ain, Awais Mehmood Kamboh, Hammad Tanveer Butt, Saima Shafait, Wasim Alamgir, Didier Stricker, Faisal Shafait

**Affiliations:** ^1^College of Computer Science and Engineering, University of Jeddah, Jeddah, Saudi Arabia; ^2^School of Electrical Engineering and Computer Science, National University of Sciences and Technology (NUST), Islamabad, Pakistan; ^3^Department of Computer Science, University of Kaiserslautern, Kaiserslautern, Germany; ^4^College of Electrical and Mechanical Engineering, National University of Sciences and Technology (NUST), Islamabad, Pakistan; ^5^Pak-Emirates Military Hospital, Rawalpindi, Pakistan; ^6^German Research Center for Artificial Intelligence (DFKI), Kaiserslautern, Germany; ^7^Deep Learning Laboratory, National Center of Artificial Intelligence, Islamabad, Pakistan

**Keywords:** open-source EEG dataset, automated EEG analytics, pre-diagnostic EEG screening, computer aided diagnosis, computational neurology, convolutional neural networks, deep learning, generalization performance

## Abstract

Electroencephalogram (EEG) is widely used for the diagnosis of neurological conditions like epilepsy, neurodegenerative illnesses and sleep related disorders. Proper interpretation of EEG recordings requires the expertise of trained neurologists, a resource which is scarce in the developing world. Neurologists spend a significant portion of their time sifting through EEG recordings looking for abnormalities. Most recordings turn out to be completely normal, owing to the low yield of EEG tests. To minimize such wastage of time and effort, automatic algorithms could be used to provide pre-diagnostic screening to separate normal from abnormal EEG. Data driven machine learning offers a way forward however, design and verification of modern machine learning algorithms require properly curated labeled datasets. To avoid bias, deep learning based methods must be trained on large datasets from diverse sources. This work presents a new open-source dataset, named the NMT Scalp EEG Dataset, consisting of 2,417 recordings from unique participants spanning almost 625 h. Each recording is labeled as normal or abnormal by a team of qualified neurologists. Demographic information such as gender and age of the patient are also included. Our dataset focuses on the South Asian population. Several existing state-of-the-art deep learning architectures developed for pre-diagnostic screening of EEG are implemented and evaluated on the NMT, and referenced against baseline performance on the well-known Temple University Hospital EEG Abnormal Corpus. Generalization of deep learning based architectures across the NMT and the reference datasets is also investigated. The NMT dataset is being released to increase the diversity of EEG datasets and to overcome the scarcity of accurately annotated publicly available datasets for EEG research.

## 1. Introduction

Neurological disorders are among the major causes of disability and death worldwide and place a significant burden on the global health system. Studies published recently (Feigin et al., 2017, 2019) indicate that neurological disorders were the leading cause-group of disability-adjusted life-years (DALYs) which is a metric employed to measure the overall number of years lost due to ill-health, disability, or early death. The global median of the total neurological workforce (including neurologists, neurosurgeons and child neurologists) is 3.1 per 100,000 population (WHO and World Federation of Neurology, 2017); consequently, reliable technological solutions that can assist in reducing the load currently placed on the neurological workforce are needed. This need is quite desperate in low-income countries where the median total neurological workforce currently stands at a dismal 0.1 per 100,000 population (WHO and World Federation of Neurology, 2017). Electroencephalogram (EEG) is a non-invasive method used to record the brain's spontaneous electrical activity over a period of time. Signals are collected by mounting a certain number of electrodes (e.g., 32, 64, 128) on the scalp according to the standard montages (Chatrian et al., 1985). It is used widely in medical practice as an inexpensive tool for diagnosis of neurological disorders and observing patterns in various medication conditions due to excellent temporal resolution as compared to other brain imaging techniques such as magnetic resonance imaging (MRI) and computed tomography (CT). The low maintenance and hardware costs of EEG make it an appealing tool for providing neurological care to patients in low-income countries. Clinically, it is generally employed as the standard test for diagnosis and characterization of epilepsy and prognostication of patients in intensive care (Yamada and Meng, 2012; Tatum, 2014). Most hospitals and clinics now generate EEG data in digital formats; if this data is curated, labeled, and stored, then the resulting repositories can be very useful for training automated EEG analytic tools that can eventually be employed to assist neurologists and physicians in providing better care to patients with neurological disorders.

Deep neural networks have received a lot of attention over the last decade and have been the primary tool of choice for automation in several application areas, including biomedical engineering. In EEG applications deep neural networks have been employed for emotion recognition (Zhang et al., 2020) and motor imagery classification (Wu et al., 2019). However, deep neural networks are known to be data hungry and require a significant amount of labeled data for training. Unfortunately, most of the EEG data generated by hospitals is either discarded or is not saved in a well-curated repository. Recently some efforts have been made to build large repositories of EEG data; one of the largest repositories of EEG data is the Temple University Hospital (TUH) dataset (Obeid and Picone, 2016). This work introduces our efforts to contribute to the cause of high-quality repositories of EEG data. Our repository is called the “*NMT*” (**N**UST-**M**H-**T**UKL EEG) dataset. At this time, the NMT dataset is divided into normal and abnormal EEG records and can be used for training to identify two classes, i.e., patients with normal and abnormal EEG. This dataset is open-source, consisting of 2,417 recordings from unique patients (1,608 male, 808 female, 1 gender unknown) spanning around 625 h. There are 2002 normal EEG recordings and 415 abnormal EEG recordings in NMT dataset version 1.0. More data is continuously being added and our plan is to release more data with future versions of the dataset.

Data labeling was performed by a team consisting of two qualified Neurologists, assisted by a technician at Military Hospital, Rawalpindi. More detailed labeling of EEG records is currently underway and will eventually be added to the repository. This dataset adds diversity to the existing public repositories of EEG data and will contribute to improving the generalization performance of analytic solutions designed for EEG. We would like to emphasize here that lack of diversity in datasets can severely limit the generalization performance of deep learning algorithms. We provide evidence of this by demonstrating severe degradation in classification performance on the NMT dataset when deep neural networks are exposed to only the TUH dataset during training and vice versa.

In addition to providing a repository of EEG data we also compare the performance of state-of-the-art deep learning algorithms on the task of EEG abnormality classification on the NMT and the TUH datasets. These algorithms can be employed for pre-diagnostic screening of normal and abnormal EEGs in under-serviced areas where neurological workforce is not available. Source-code for all our experiments is available in a publicly accessible GitHub repository (link available at: https://dll.seecs.nust.edu.pk/downloads/). This source-code is shared to ensure that our research is transparent and reproducible. Furthermore, it will also help deep learning and EEG researchers quickly generate baseline results on the NMT, TUH (and other) EEG data repositories.

The primary contribution of this work is the NMT EEG dataset consisting of 2,417 anonymized EEG recordings containing around 625 h of data is shared in the public domain. Each recording in the NMT dataset is labeled as either normal or abnormal (pathological) by a team of expert neurologists. Furthermore, the following experiments have also been conducted:

- Validation of the NMT dataset is achieved by comparing classification performance of deep learning algorithms on this dataset with the baseline achieved on the existing TUH EEG dataset.
- Performance of state-of-the-art deep learning algorithms by Schirrmeister et al. (2017b) and Roy et al. (2019) is investigated at the task of classification of EEG records as normal or abnormal, using the NMT dataset. For the purpose of reproducibility of results presented here, the code for the deep learning algorithms we used for classification of normal and abnormal EEG records is shared in the public domain.
- Preliminary results are presented on the impact of variation in data sources on the generalization performance and transfer learning of the algorithms and datasets. To the best of our knowledge, this is the first study of its kind to investigate model bias in classifying normal/abnormal EEG.

The rest of this paper is organized as follows: The salient features of the NMT dataset and the data collection and labeling protocols are described in section 2. The problem of pre-diagnostic screening of normal and abnormal EEGs is introduced in section 3; this is followed by a description of the various deep learning approaches employed for this problem. Results and discussion are presented in sections 4 and 5, respectively. Conclusions are presented at the end in section 6.

## 2. The NMT DataSet

Availability of a large repository of data from numerous sources is critical for the development of robust analytic solutions. Building such a repository is quite difficult and requires investment of significant effort, time, and financial resources. The TUH corpus (Obeid and Picone, 2016) is one of the few existing publicly available datasets that is large enough for training large-scale deep neural networks. This dataset, although quite extensive, consists of records from only a single hospital. We believe that availability of data from more hospitals will be beneficial for development of robust analytic solutions for EEG applications since, such data will expose learning algorithms to variations introduced by different acquisition hardware, data recording protocols, and population demographics. To the best of our knowledge, the NMT is the only open-source EEG dataset collected from a South-Asian demographic.

### 2.1. Data Collection Protocol

Data collection for this work was done at the Pak-Emirates Military Hospital (MH), Rawalpindi, Pakistan. Details of project proposal were submitted to the hospital's institutional review board (IRB) for review and data collection began after the IRB consented to approve the project (IRB number 51214MH, dated March-15-2019). The hardware used for data collection was the KT88-2400 system manufactured by Contec Medical Systems. Recording sessions were conducted between 11:00 and 17:00 h. All adult patients were advised to get a full night's sleep before the recording session. For children aged 2 years or less, recording sessions were conducted while they were asleep. All patients were instructed to avoid taking any sedatives or sleep medication before coming in for a recording session. Patients on anti-epileptic drugs were instructed to continue consuming them according to their prescription. All patient identity information was removed before uploading the EEG records to the project database. Before recording sessions, patients were given a consent form containing a summary of the project and asking whether they would consent to contributing their EEG data for the project repository. This form was available both in English and in Urdu (Pakistan's national language). Patients who consented to contributing their data provided written approval by signing the consent forms. Our target during this project is to use the NMT dataset for training machine learning algorithms that can be employed for screening of normal and abnormal EEGs. Such screening tools could possibly be deployed in rural areas of Pakistan (and other developing countries) to identify patients in need of neurological care and forwarding their cases to neurologists in larger hospitals in cities for further examination and consultation. EEG recording session were administered by a qualified technician with 5 years of experience of managing the hospital's EEG recording room. Each EEG record was marked as either normal or abnormal by the neurological staff of hospital trained in EEG interpretation. To improve the intra-rate-agreement, this data was then forwarded to two expert neurologists, who either accepted or modified the label assigned by the staff. Both neurologists had to agree on a label before it was included in the dataset. In case of disagreement, between the neurologists, the record in question was not included in the dataset.

### 2.2. Data Statistics

**Figure 1:**
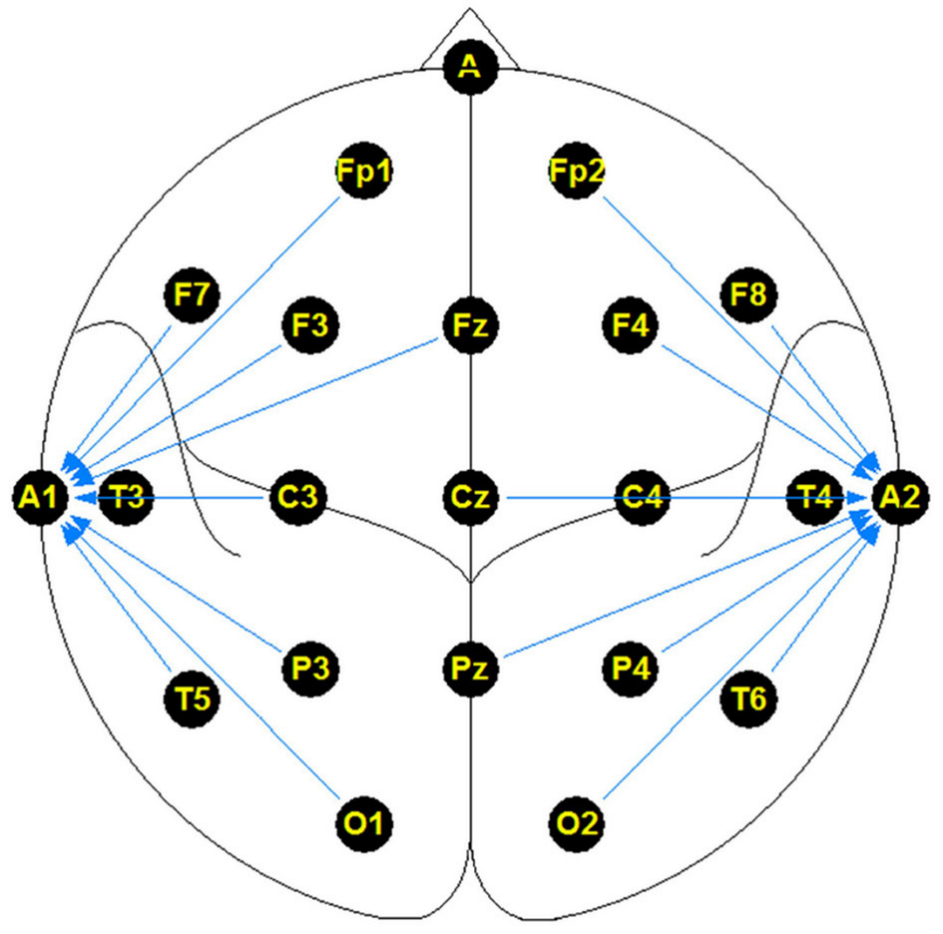
Linked ear referenced standard electrode montage.

**Figure 2:**
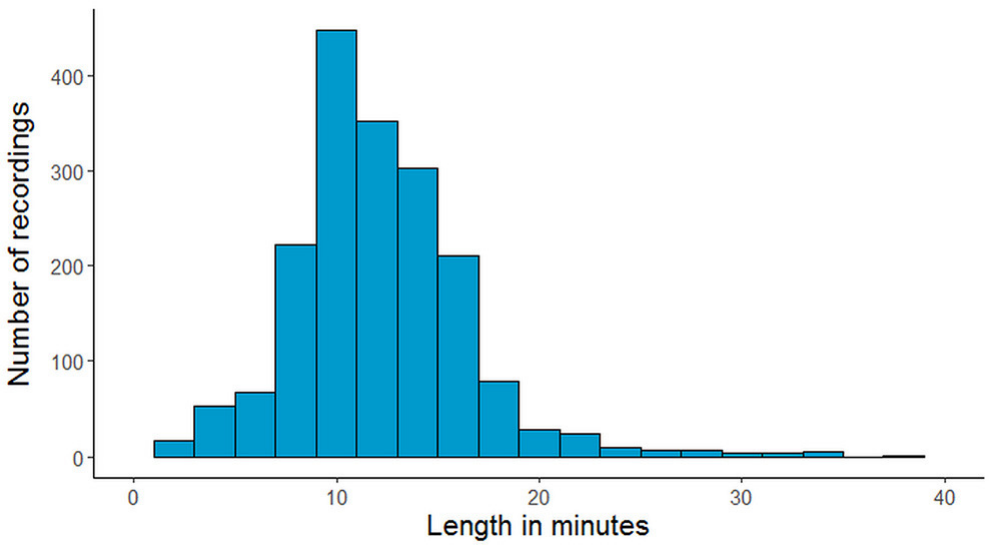
The number of recordings in the NMT dataset for each range of duration in minutes.

**Figure 3:**
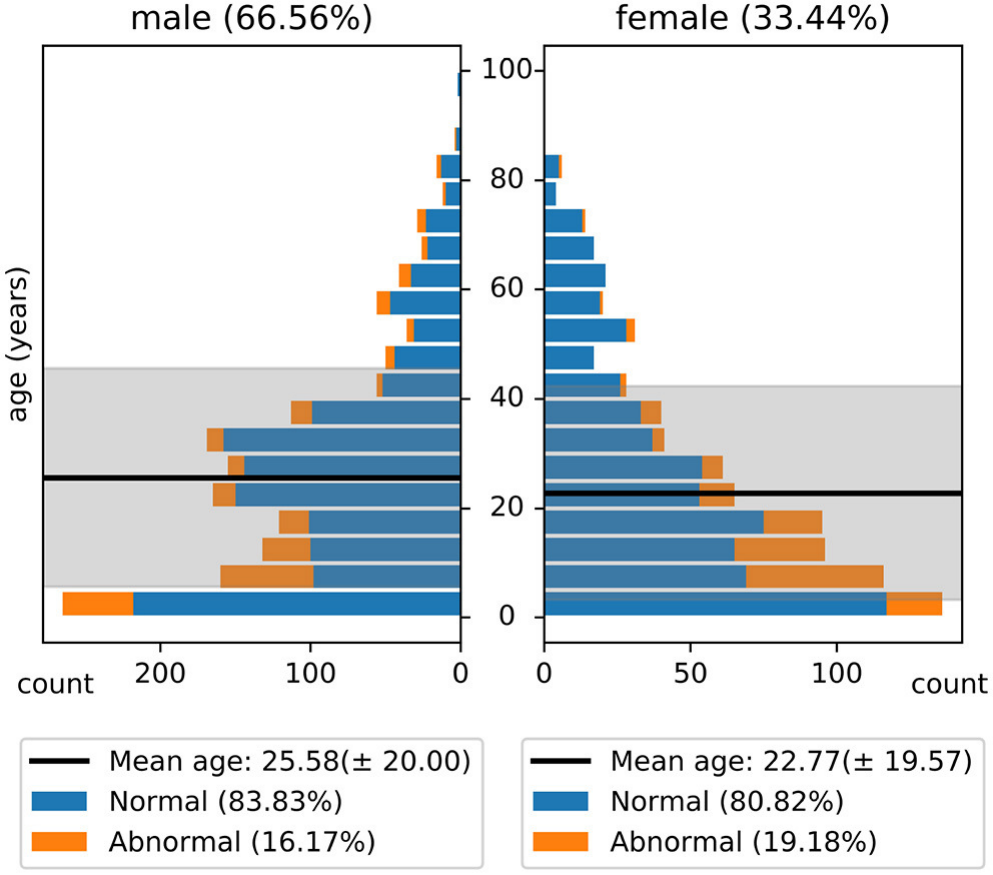
Histogram of age distribution in the NMT dataset. The shaded regions indicate the standard deviations of the age of male and female subjects in the dataset.

The NMT dataset consists of 2,417 EEG records at this time. The EEG montage used for recordings consists of the standard 10-20 system and is shown in Figure 1. There are 19 channels on the scalp, channels A1 and A2 are reference channels on auricle of the ear. The sampling rate of all channels is 200 Hz. The average duration of each record is 15 min. The histogram of recording lengths is given in Figure 2. The histograms of age distributions of males and female subjects in the dataset are shown in Figure 3. The age ranges from under 1 year old up to 90 years old; 66.56 and 33.44% of the records are collected from male and female subjects, respectively. 16.17% of EEG recordings from males are abnormal/pathological whereas, in case of females, 19.18% records are abnormal/pathological.

### 2.3. Pre-processing

NMT data set is acquired using standard linked ear reference at sampling rate of 200 Hz. A linked ear reference means that the electrodes on the ears are linked together and serve as the reference for the signals recorded from all other electrodes. Although there is no significant superiority of one montage over others, we re-referenced the NMT dataset offline to average reference for comparability with TUH dataset, resulting in 21 EEG channels for each record. Pre-processed recordings consist of average referenced channel signals in European Data Format (EDF).

### 2.4. Dataset Structure

The EEG records are available in the open-source EDF format. The directory structure of the NMT dataset is as follows.

1. ./abnormal: This directory contains all EEG records labeled as “abnormal” by the team of neurologists. Files within in this directory are organized under two sub-directories (1) “./abnormal/train” which contains all abnormal EEG records that were used for training in all our experiments and (2) “./abnormal/eval” which contains all abnormal EEG records that were used for evaluating the performance of the algorithms discussed in this paper. This division is provided to allow reproducability of presented results.
2. ./normal: This directory contains all EEG records labeled as “normal” by the team of neurologists. Files within in this directory are organized in the same manner as in the abnormal directory.
3. Labels.csv: This file contains a list of the EEG records along with demographic information and the ground-truth label. Below is a brief description of each column in this file.

(a) recordname: This column contains the name of each record.
(b) label: The ground-truth label assigned to the record by the team of neurologists. This column contains one of two labels: “normal” and “abnormal.”
(c) age: Age of the patient (in years).
(d) gender: Gender of the patient. This column contains one of three labels: “male,” “female,” or “not specified.”
(e) loc: Location of the file, indicating if this record is included in the “training” or “evaluation” set.

For example, record 0000024.edf has label = normal and loc = eval; it means that this record may be found in the “./normal/eval” directory. Similarly, the record 0000025.edf has label = abnormal and loc = train; meaning that this record can be found in the “./abnormal/train” directory.

4. DataStat.py: This is a python script which can be used to plot the population Pyramid of the demographic information given in the “Labels.csv” file and saves it as a .png image. This file can also be employed to examine different statistics about the dataset. The authors plan to continue adding records to the NMT dataset and this script maybe useful for obtaining statistics of future iterations of the dataset.

The EDF file format, apart from the physiological data, includes related information such as channel names and number of channels, the sampling rate and the low/high cut off frequencies of the bandpass filters. The date and time mentioned in the files correspond to the time when the files were saved in this particular format, and do not relate to the time of the recording.

## 3. Pre-diagnostic Screening of EEG

As mentioned previously, the median neurological workforce in low-income countries is 0.1 per 100,000 population. This means that one member of this workforce must provide service to one million people. In 2013, for example, there were only 134 qualified neurologists in Pakistan (Siddiqui et al., 2015), a country of 212 million people. Furthermore, most of these qualified doctors are concentrated in only three major cities of the country. Non-availability of qualified neurologists generally leads to patients in remote areas receiving little to no neurological care. In these conditions, automated EEG screening tools can be invaluable in providing at least some form of care to patients in under-served areas. Coupled with custom-built, low-cost EEG hardware these tools can be used to perform pre-diagnostic screening of normal and abnormal EEG so that patients with abnormal EEGs can be referred to neurologists for more detailed examination and investigation. However, to ensure reliable performance these screening tools must be trained on diverse and well-curated EEG datasets.

There have been some attempts dedicated to classification of normal and abnormal EEG; these are listed in Table 1. López et al. (2017) used handcrafted features. Pre-trained models were used in Amin et al. (2019) and Alhussein et al. (2019). Apart from Roy et al. (2019) and Gemein et al. (2020), all approaches evaluated only CNN based architectures. With the exception of van Leeuwen et al. (2018), all these approaches were trained using the TUH Abnormal EEG corpus^1^ which consists of 2,978 EEG recordings (1,506 normal and 1,472 abnormal). The most extensive evaluation of this task was conducted in Gemein et al. (2020) where multiple (conventional) handcrafted and (deep learning based) end-to-end architectures were tested. Among the approaches listed in Table 1 we tested three (using publicly available code) on the NMT data and examined whether we could generate results similar to those reported on the TUH data. There were multiple reasons for using existing deep learning approaches.

- First, achieving classification accuracy similar to an existing, well-reputed, dataset verifies the general correctness of the new dataset.
- Second, this exercise enables generation of baseline results for comparison in this and future extensions of our work.
- Third, reproduction of results in reference papers by a third party (us) contributes to the cause of reproducible research.
- Fourth, implementation of multiple approaches enables performance comparison and allows us to rank existing algorithms based on their performance on the NMT dataset.

**Table 1:** Related works on classification of normal/abnormal EEGs based on the TUH Abnormal EEG Corpus.

**Automated diagnosis**	**Architecture**	**Accuracy**
López et al., 2017	CNN + MLP	78.8
Schirrmeister et al., 2017a	Deep CNN	85.4
Roy et al., 2019	ChronoNet	86.6
Amin et al., 2019	AlexNet + SVM	87.3
Alhussein et al., 2019	3 x AlexNet + MLP	**89.1**
Gemein et al., 2020	RG	85.9
Gemein et al., 2020	BD-TCN	86.2
van Leeuwen et al., 2018	Deep CNN	82.0

*van Leeuwen et al. (2018) did not use TUH dataset. Boldface indicates highest value*.

Following the same convention as the TUH abnormal corpus, we extracted an independent, test set which was used for final performance evaluation of a network architecture after learning parameter using the training and validation folds.

### 3.1. ChronoNet

The ChronoNet architecture was purpose-built for EEG data analysis (Roy et al., 2019); it uses recurrent neural networks (RNNs) and was inspired by state-of-the-art image classification techniques like inception (Szegedy et al., 2015) and dense connections (Huang et al., 2017). It uses inception layers with exponentially varying kernel lengths for 1D convolution layers in combination with densely connected recurrent layers. It was experimentally demonstrated that exponentially varying filter lengths enabled the network to extract information over multiple timescales and lead to improved performance. It was surmised that in the EEG time series data, the range of timescales in which features exist was much wider compared to those captured in the images. Roy et al. (2019) reported that they were able to classify normal and abnormal EEG records, from the TUH dataset, with an accuracy of 86.57%. The Temporal Central parietal (TCP) montage was used for all experiments in Roy et al. (2019). We employed referencing and pre-processing techniques that were identical to Roy et al. (2019) in all experiments that used the ChronoNet architecture. The ChronoNet architecture was trained using the open-source implementation developed by Patel et al. (2018). Results are presented in section 4.

### 3.2. Deep and Shallow CNNs

The “Deep” and “Shallow” CNN architectures were proposed in Schirrmeister et al. (2017b) with the objective to customize CNN based architectures, typically used for image analysis, for decoding and analysis of EEG data. However, attempts to incorporate domain knowledge into deep learning architectures can be counter-productive as well, since they can easily turn into handcrafting which goes against the data-driven principles that lie at the core of deep learning. Consequently, some caution needs to be exercised when customizing deep learning architectures for applications. The Deep CNN architecture consists of a special first block that is designed to handle EEG data; it works by applying convolution twice: first across time and then across the EEG channels. This block is followed by three blocks of standard convolution and max-pooling layers. The final layer is a fully connected, dense layer that uses softmax functions for classification. This architecture employs exponential linear units (ELUs) as activation functions. ELUs use the following activation function:


(1) $$\begin{array}{r} f\left(x\right)=\{\begin{array}{ll} x & \forall \;x>0\\ e^{x}-1 & \forall \;x\leq 0 \end{array} \end{array}$$

Compared to the Deep CNN architecture, which is rather generic, the shallow CNN architecture is tailored to learn band-power features. It employs pre-designed spatial filters and temporal filters inspired from the filter bank common spatial patterns (FBCSP) approach first presented in Ang et al. (2008). This is followed by a squaring function, a mean pooling layer and a logarithmic activation function.

Just like the Deep CNN model, it uses ELUs as activation functions and alternating convolution-and-pooling layers. Maximum overlapping crops are used for capturing time dependencies. By maximum overlapping we mean that adjacent crops had only one non-overlapping time sample. The ConvNet parameters are optimized using stochastic gradient descent with the Adam optimizer. Maximum overlapping crops were used for capturing time dependencies where crop-wise training (Schirrmeister et al., 2017a) forced models to learn the anomalies rigorously and were shown to be effective by the authors. We trained the models by using the Braindecode library developed by Schirrmeister et al. (2017b). The minimal pre-processing techniques of downsampling, clipping voltage, and scaling were used in same fashion.

### 3.3. Hybrid Deep CNN and LSTM

**Figure 4:**
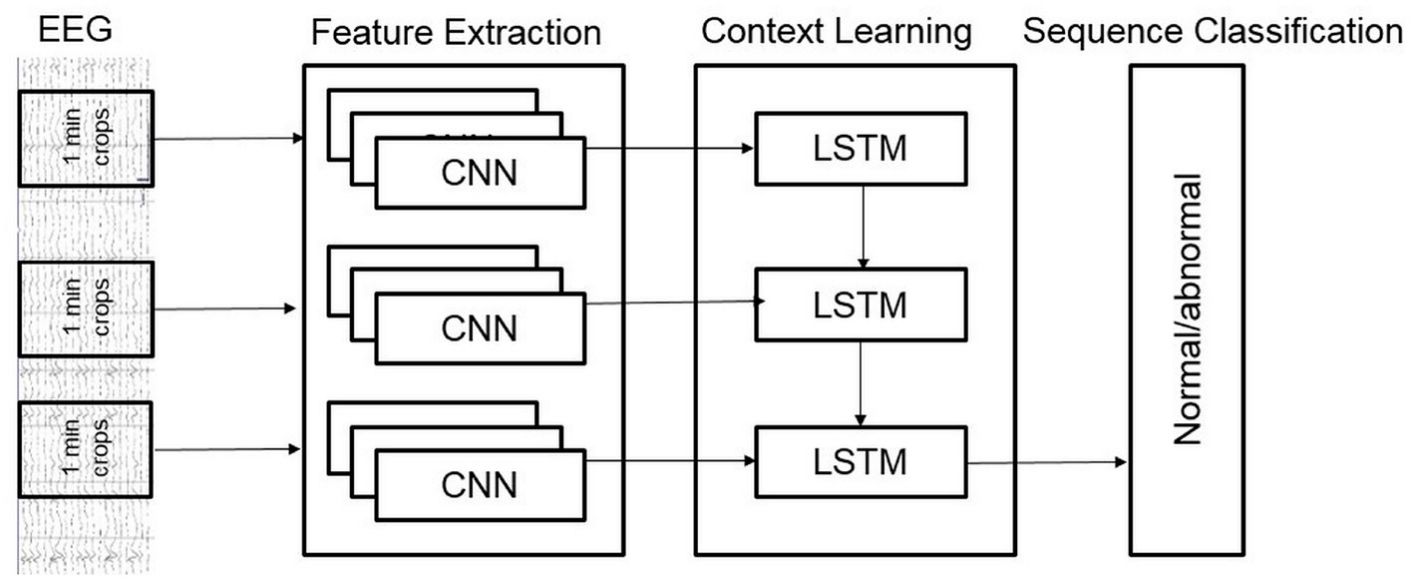
Architecture of our hybrid model.

We also developed a novel hybrid model that added a layer of Long Short-Term Memory (LSTM) units on top of the Deep CNN architecture. Our hybrid architecture is illustrated in Figure 4; it treats the Deep CNN architecture as a “Feature Extractor.” This model is obtained by removing the final softmax layer of the Deep CNN architecture and then taking features from all 1-min windows of a recording and feeding them to an LSTM for sequence classification. The Deep CNN model of Schirrmeister et al. (2017b) uses simple statistical averaging of classifier scores over short, time windows to obtain a final label for the whole EEG record. In contrast, our hybrid architecture uses LSTMs to make decisions by taking into account temporal dependence between windows spaced far apart from each other. The motivation behind the hybrid architecture is to see if replacing the simple averaging based decision making (of the Deep CNN) with a more learnable approach (introduced by the LSTMs) delivers an improvement in performance.

### 3.4. Fine-Tuning

Fine-tuning (or transfer learning) is a technique that is often employed when moving across applications or datasets (Tan et al., 2018). A typical deep neural network has millions of learnable parameters that are learnt by feeding it a large number of labeled training examples. Unfortunately, building large-scale repositories of labeled training examples for medical applications is an expensive exercise. Therefore, researchers in the medical domain often take a deep neural network that has been pre-trained on a larger size dataset, often from a non-medical application, and fine-tuning its parameters on the relatively smaller (application specific) medical dataset. For example, in medical imaging applications a deep CNN is first trained on millions of natural images and then fine-tuned on the relatively smaller dataset of medical images in most applications (Shin et al., 2016).

Fine-tuning often works because the vast majority of learnable parameters of the network are learnt during the initial training on the larger dataset; only a small subset of parameters need to be learnt during the fine-tuning phase and the smaller application specific dataset is often sufficient for this purpose. In some applications fine-tuning is also performed across two datasets from the same domain as well. This type of approach can help to ameliorate the impact of variation in data sources and acquisition devices. To investigate whether this hypothesis is valid in the EEG domain as well we examined the efficacy of fine-tuning across the TUH and NMT datasets. More specifically, we first trained the Shallow and Deep CNN architectures from scratch on the, larger, TUH dataset. Training from scratch means that the weights of all layers were initialized to random values and then learned using the TUH dataset. After training the network from scratch on the TUH dataset we fine-tuned its weights using the NMT dataset. This was done by starting with the weights learned by training on the TUH dataset and updating them using the data in the NMT dataset. Early stopping was employed to monitor the loss function on the (NMT) training and validation data to ensure overfitting was avoided. In general, fine-tuning on a certain dataset requires a smaller number of epochs compared to training it from scratch on the same dataset.

### 3.5. Performance Evaluation Metrics

We employed three distinct metrics to evaluate the performance of different algorithms in our experiments. These metrics are summarized below:

- Accuracy: Accuracy is defined as the total number of EEG records correctly predicted as normal/negative or abnormal/positive, divided by the total number of EEG records.

(2) $$\begin{array}{r} \text{Accuracy}=\frac{TP+TN}{TP+TN+FN+FP} \end{array}$$

Where, *TP* denotes the number of true positives, *TN* denotes the number of true negatives, *FN* denotes the number of false negatives and *FP* denotes the number of false positives.

- F1-score: The F1-score is defined as the harmonic mean of *precision* and *recall*.

(3) $$\begin{array}{r} \text{F1}=2\times \frac{\text{precision}\times \text{recall}}{\text{precision}+\text{recall}} \end{array}$$

Where, *precision* = *TP*/(*TP*+*FP*) and recall = *TP*/(*TP*+*FN*).

- AUC: The AUC represents the area under the ROC-curve and varies between the lowest value of the 0 and the highest value of 1. AUC is often employed to evaluate the performance of binary classification and tends to work well even when class distributions are unbalanced.

## 4. Experiments and Results

Performance of the different architectures described in section 3 was individually tested on both the TUH and the NMT datasets for comparison purposes. We also examined the generalization performance of different architectures across the two datasets. Out of the total 2,417 recordings, a set of 185 recordings (90 abnormal and 95 normal) have been set aside as an independent “Test” (or evaluation) set. The purpose of keeping a fixed test set is to allow future users the ability to compare the performance of their work with the algorithms reported here and in subsequent research. For the purpose of all experiments in this paper, the performance was evaluated on the independent test set. The remaining set of 2,232 recordings was further subdivided into “Training” and “Validation” folds, using a 90–10% split. Each network was trained in two phases. During phase-I, a network was trained until accuracy on the validation fold saturated and did not improve for a predefined number of epochs. At this point, the learnt model was saved, the value of the loss function was noted and training and validation folds were merged. In phase-II, the model saved at the end of phase-I was reloaded and training recommenced on the entire training set until the loss function dropped back to the value it was at when phase-I ended. Training was then stopped and performance was evaluated on the, previously unseen, independent test set. The majority of files in the TUH dataset have a sampling rate of 250 Hz. However, it also contains some files sampled at 256, 400, and 512 Hz. Therefore, for the purpose of all experiments in this paper all EEG records (from both TUH and NMT datasets) were downsampled to 100 Hz before training and testing.

### 4.1. Baseline Implementation

The first set of experiments conducted were to reproduce the results of existing deep learning architectures on the benchmark TUH dataset. Matching the performance reported in reference work validated our implementation and allowed us to compare the performance of different approaches on the task of pre-diagnostic EEG screening. Furthermore, once we were able to match the reported performance on the TUH dataset, we repeated the same learning protocol on the NMT dataset to evaluate whether we could obtain similar performance. The performance obtained for different architectures on the TUH and the NMT datasets are presented in Tables 2, 3, respectively. The accuracy achieved by ChronoNet on the TUH dataset was reported to be 86.75% in Roy et al. (2019); the highest accuracy that we obtained in our implementation of this architecture was lower at 81% resulting in a noticeable gap between the results reported by Roy et al. (2019) and what we observed. This performance gap could possibly be due to small variations in the experimental setup between our implementation and the reference implementation. This architecture was able to obtain an accuracy of around 76% on the NMT dataset. The accuracies reported in Gemein et al. (2020) on the TUH dataset using the Shallow and Deep CNN architectures are 84.1 and 84.6%, respectively; our implementations of these architectures delivered similar performance on the TUH dataset (accuracy = 84%). When we tested these architectures on the NMT dataset, the Deep CNN architecture demonstrated an accuracy of 77%, whereas the performance of the Shallow architecture was slightly lower at 72%. In case of the shallow architecture the degradation in performance on the NMT dataset could possibly be due to the reliance of the shallow architecture on a handcrafted approach. In case of the Deep CNN the performance degradation could be due to the smaller size of the NMT dataset in comparison to the TUH dataset. The hybrid approach delivered an accuracy of 85% on the TUH dataset and 79% on the NMT dataset.

**Table 2:** Performance of different architectures on the TUH dataset.

**Architecture**	**F1-score**	**Accuracy**	**AUC**
ChronoNet	0.78	0.81	0.83
Shallow-CNN	0.82	**0.85**	0.93
Deep-CNN	0.82	0.84	0.92
Hybrid	**0.84**	**0.85**	**0.94**

*Boldface indicates highest value*.

**Table 3:** Performance of different architectures on the NMT dataset.

**Architecture**	**F1-score**	**Accuracy**	**AUC**
ChronoNet	0.75	0.76	0.77
Shallow-CNN	0.70	0.72	0.72
Deep-CNN	0.77	0.77	0.84
Hybrid	**0.78**	**0.79**	**0.86**

*Boldface indicates highest value*.

### 4.2. Generalization Performance and Fine-Tuning

In actual deployment scenarios any automated screening algorithm is highly likely to be presented with data from unseen sources. It is therefore, critical to examine the generalization performance of different architectures to investigate whether they are robust to variations in acquisition devices and sources. Ideally, the performance of deep learning algorithms should remain consistent from one dataset to another. However, this is not always observed. Evaluating performance of abnormal EEG detection across datasets has not been possible so far since there was only one publicly available dataset specifically for this problem. We evaluated the generalization performance of the Deep and Shallow CNN architectures by training them on the TUH dataset and then evaluating performance on the NMT dataset. We observed that there was a noticeable degradation in performance with accuracy and AUC reducing to 45% and 0.48, respectively for the Shallow CNN architecture and 48% and 0.46 for the Deep CNN architecture. A similar degradation in performance was observed when these architectures were trained on the NMT dataset and tested on the TUH dataset. These results highlight the need for collection of diverse datasets from multiple sources since algorithms trained on data from only a single source do not generalize well in the case of EEG data.

The results for fine-tuning are presented in Table 4. To the best of our knowledge, these are the first results of fine-tuning across multiple EEG datasets. When compared with the baseline setup (Table 3), in which a network is trained and tested on the same dataset; fine-tuning across datasets delivers a performance gain in case of the Deep CNN architecture. However, no gains are observed for the Shallow CNN architecture. The number of parameters in the Shallow CNN architecture is quite small and no performance gain after fine-tuning is not surprising. In case of the Deep CNN architecture which has more learnable parameters, the gains delivered by fine-tuning are substantial and illustrate the benefit of exposing deep learning architectures to multiple datasets.

**Table 4:** Performance of CNN architectures fine-tuned on the NMT dataset after training on the TUH dataset.

**Architecture**	**F1-score**	**Accuracy**	**AUC**
Shallow-CNN	0.70	0.71	0.82
Deep-CNN	0.82	0.82	0.87

## 5. Discussion

We have presented a new publicly accessible repository of EEG data, collected specifically for development of data analytic tools. The suitability of this dataset for deep learning applications was investigated in detail. Several existing deep learning architectures were used for performance analysis on a sample task of pre-diagnostic screening of abnormal EEG records. Comparative analysis demonstrated that CNN based architectures and the Hybrid architecture delivered the best performance on the TUH dataset. On the NMT dataset we were able to obtain similar performance trends, with scores obtained for each architecture being slightly lower than the corresponding scores on the TUH dataset. The degradation in performance of all architectures when applied to the NMT dataset can be attributed to the following factors:

1. The number of normal and abnormal records in the TUH dataset is more or less balanced. In contrast, only about 15% (325) of the records in the NMT training set are abnormal. This means that a network trained only on the NMT dataset is exposed to a comparatively smaller number of abnormal records. Therefore, lower performance compared to a dataset with more abnormal examples is not unexpected. We did not take any measures to suppress the unbalanced distribution of the NMT data because we want it to reflect the natural frequency of abnormalities within the population. This naturally occurring unbalanced distribution data distribution is more realistic and also more challenging to deal with. It also highlights the need for development of novel data augmentation and (synthetic) generation strategies which are commonly used in computer vision applications but have been investigation in the EEG context only recently (Luo and Lu, 2018; Lashgari et al., 2020).
2. Different demographics can also be a potential contributing factor. The NMT dataset contains data from a relatively younger population (average age = 24.64 years) whereas the TUH abnormal dataset contains data from an older population (average age = 49 years).
3. Use of different hardware for data collection is also another factor that can have an impact on the results. The NMT and TUH datasets were collected using different EEG acquisition devices. Therefore, small differences in signal characteristics could have contributed to the difference in performance.

Taking the above factors into account, the performance gap is not surprising however, the results in Table 3 are close enough to the reference dataset to give us confidence about the quality of the NMT dataset. In addition to the factors listed above, one may argue that differences in the training protocol of the deep learning architectures could also be a contributing factor. This argument might be valid in case of the ChoronoNet architecture (Patel et al., 2018) since the original authors did not publicly release their code. However, we believe that this is unlikely in the case of the Shallow and the Deep CNN architectures (Schirrmeister et al., 2017b; Gemein et al., 2020) since we used the libraries provided by the original authors and closely followed the guidelines provided in their work. We also want to highlight that we are working on adding more data to the NMT dataset and are confident that this performance gap will reduce as the size of our dataset increases as part of future updates. An interesting research direction can be to explore whether data augmentation and generation strategies can bridge the performance gap between the unbalanced NMT dataset and the balanced TUH dataset.

In order to evaluate the generalization performance of deep learning algorithms we evaluated their performance on EEG data sources which they were not exposed to during training. Algorithms trained on only the TUH data and tested on the NMT data demonstrated degradation in performance. A similar degradation was observed when training was performed using the NMT data and testing was done using the TUH data. This indicates that despite achieving high classification performance on individual datasets, the performance of current deep learning algorithms degrades when applied to data from different sources. This also implies that prior to being deployed in real life scenarios, these algorithms must be extensively trained and tested on data from multiple sources. We believe that the NMT dataset can play a role in enabling the development of robust deep learning based EEG analysis tools. More detailed analysis is required to further investigate the underlying reasons behind the degradation in performance.

Fine-tuning on NMT data after initial training on TUH data delivered a noticeable performance gain in case of the Deep CNN architecture whereas, no performance gain was observed for the Shallow CNN architecture. This is not surprising since the Deep CNN has more tunable parameters, compared to the Shallow CNN, and thus benefits from exposure to more data. With the creation of NMT dataset, the research community is in a position to conduct detailed examinations of the generalization and fine-tuning performance across different EEG datasets. We expect such studies to provide valuable insights about the application of deep learning to EEG data analysis.

## 6. Conclusions

We have presented the NMT dataset which consists of a large repository of EEG recordings labeled as normal and abnormal. At this time, the NMT dataset can be employed to train machine learning algorithms for pre-diagnostic screening of normal and abnormal EEG recordings. The performance of deep learning architectures was verified using this new dataset. Furthermore, we have also investigated the generalization performance of these approaches. Our analysis indicates that existing deep learning approaches work well when trained and tested on data from the same source(s) but their performance degrades significantly when they are tested on data sources to which they don't have any prior exposure. Consequently, there is a need to simultaneously collect more extensive and diverse datasets and to develop robust deep learning algorithms that can handle variations in data sources and acquisition devices. Preliminary analysis also indicates that fine-tuning delivers performance gains when applied across different EEG datasets. We hope that this work will motivate researchers to examine the generalization performance and fine-tuning of deep learning models on EEG data in more detail as an important future direction.

## Data Availability Statement

The datasets presented in this study can be found in online repositories. The names of the repository/repositories and accession number(s) can be found below: https://dll.seecs.nust.edu.pk/downloads/.

## Ethics Statement

The studies involving human participants were reviewed and approved by Institutional Review Board of the Pak-Emirates Military Hospital, Rawalpindi, Pakistan. Written informed consent to participate in this study was provided by the participants' legal guardian/next of kin.

## Author Contributions

RU and HB implemented all deep learning architectures, conducted all experiments for this paper, and wrote the first draft of the paper. HK edited subsequent drafts of the paper. HK, SS, and WA facilitated data collection, labeling and organization for this work, and helped with obtaining IRB approvals. SS and WA provided neurological expertise and supervised the data labeling team. AK, DS, and FS developed methodologies for the technical parts of this paper and supervised the experiments conducted by RU and HB. The final draft of this paper was edited jointly by AK, DS, and FS. All authors contributed to the article and approved the submitted version.

## Funding

This work was funded by the University of Jeddah, Jeddah, Saudi Arabia, under grant No. (UJ-21-ICI-1). The authors, therefore, acknowledge with thanks the University of Jeddah technical and financial support. The data collection and deep learning part of work was initially supported by DAAD (German Academic Exchange Service); project number 57459097 under TUKL-NUST partnership. The authors would therefore, also like to thank the DAAD and TUKL Germany for their technical and financial support.

## Conflict of Interest

The authors declare that the research was conducted in the absence of any commercial or financial relationships that could be construed as a potential conflict of interest.

## Publisher's Note

All claims expressed in this article are solely those of the authors and do not necessarily represent those of their affiliated organizations, or those of the publisher, the editors and the reviewers. Any product that may be evaluated in this article, or claim that may be made by its manufacturer, is not guaranteed or endorsed by the publisher.

## Footnotes

^1^Amin et al. (2019) and Alhussein et al. (2019) augmented the TUH Abnormal dataset with an additional 10,000 normal recordings.
