# Corrigendum: The NMT Scalp EEG Dataset: An Open-Source Annotated Dataset of Healthy and Pathological EEG Recordings for Predictive Modeling

**DOI:** 10.3389/fnins.2022.877868

**Published:** 2022-03-15

**Authors:** Hassan Aqeel Khan, Rahat Ul Ain, Awais Mehmood Kamboh, Hammad Tanveer Butt, Saima Shafait, Wasim Alamgir, Didier Stricker, Faisal Shafait

**Affiliations:** ^1^College of Computer Science and Engineering, University of Jeddah, Jeddah, Saudi Arabia; ^2^School of Electrical Engineering and Computer Science, National University of Sciences and Technology (NUST), Islamabad, Pakistan; ^3^Department of Computer Science, University of Kaiserslautern, Kaiserslautern, Germany; ^4^College of Electrical and Mechanical Engineering, National University of Sciences and Technology (NUST), Islamabad, Pakistan; ^5^Pak-Emirates Military Hospital, Rawalpindi, Pakistan; ^6^German Research Center for Artificial Intelligence (DFKI), Kaiserslautern, Germany; ^7^Deep Learning Laboratory, National Center of Artificial Intelligence, Islamabad, Pakistan

**Keywords:** open-source EEG dataset, automated EEG analytics, pre-diagnostic EEG screening, computer aided diagnosis, computational neurology, convolutional neural networks, deep learning, generalization performance

In the published article, there was a mistake in the affiliations of Hassan Aqeel Khan “School of Electrical Engineering and Computer Science, National University of Sciences and Technology, Islamabad, Pakistan” will be removed as an affiliation for Hassan Aqeel Khan.

In the published article, there was an error in the affiliation “Department of Computer Science and Engineering College, University of Jeddah, Jeddah, Saudi Arabia.” It should be “College of Computer Science and Engineering, University of Jeddah, Jeddah, Saudi Arabia.”

There was also an error in the affiliation “School of Electrical Engineering and Computer Science, National University of Sciences and Technology, Islamabad, Pakistan.” It should be “School of Electrical Engineering and Computer Science, National University of Sciences and Technology (NUST), Islamabad, Pakistan.”

There was a mistake in the affiliation “Department of Computer Science, Technical University of Kaiserslautern, Kaiserslautern, Germany.” It should be “Department of Computer Science, University of Kaiserslautern, Kaiserslautern, Germany.”

Additionally, there was an error in the affiliation “German Research Center of Artificial Intelligence (DFKI), Kaiserslautern, Germany.” It should be “German Research Center for Artificial Intelligence (DFKI), Kaiserslautern, Germany.”

The authors apologize for these errors and state that they do not change the scientific conclusions of the article in any way. The original article has been updated.

## Publisher's Note

All claims expressed in this article are solely those of the authors and do not necessarily represent those of their affiliated organizations, or those of the publisher, the editors and the reviewers. Any product that may be evaluated in this article, or claim that may be made by its manufacturer, is not guaranteed or endorsed by the publisher.

